# A Case of Liver Abscess with *Desulfovibrio desulfuricans* Bacteremia

**DOI:** 10.1155/2015/354168

**Published:** 2015-01-08

**Authors:** Saho Koyano, Keita Tatsuno, Mitsuhiro Okazaki, Kiyofumi Ohkusu, Takashi Sasaki, Ryoichi Saito, Shu Okugawa, Kyoji Moriya

**Affiliations:** ^1^Department of Infection Control and Prevention, The University of Tokyo Hospital, 7-3-1 Hongo, Bunkyo, Tokyo 113-8655, Japan; ^2^Department of Microbiology, Gifu University Graduate School of Medicine, 1-1 Yanagido, Gifu, Gifu 501-1194, Japan; ^3^Department of Gastroenterology, The University of Tokyo Hospital, 7-3-1 Hongo, Bunkyo, Tokyo 113-8655, Japan; ^4^Department of Microbiology and Immunology, Graduate School of Health Care Science, Tokyo Medical and Dental University, 1-5-34 Yushima, Bunkyo, Tokyo 113-0034, Japan

## Abstract

*Desulfovibrio* spp. are gram-negative, sulfate-reducing, and anaerobic bacteria found in the digestive tract of humans. Because *Desulfovibrio* spp. are infrequent causative agents of infectious diseases and are difficult to isolate and to identify from clinical specimens, the appropriate antibiotic therapy to infection with *Desulfovibrio* spp. has not been determined. We report the first case of liver abscess with bacteremia due to *Desulfovibrio desulfuricans* to show the clinical presentation and treatment. The patient was successfully treated with intravenous piperacillin-tazobactam and oral amoxicillin-clavulanic acid.

## 1. Introduction

*Desulfovibrio* spp. are a group of gram-negative, nonsporulating, curved, sulfate-reducing, and anaerobic bacteria that are ubiquitously distributed in soil, water, and sewage. To date, four species of* Desulfovibrio* have been isolated from human specimens:* D. desulfuricans*,* D. fairfieldensis*,* D. vulgaris*, and* D. piger* [1, 2]. In human and animal,* Desulfovibrio* spp. colonize oral cavities and gastrointestinal tracts [3, 4] and infrequently cause infections in human, such as periodontitis, brain abscess, liver abscess, and blood stream infection [2, 5–10]. Few case reports have described the use of antimicrobials in curing* Desulfovibrio*-infected patients; additional case reports are needed to determine the antibiotic therapy appropriate for the treatment of such infections.

The difficulty of identifying* Desulfovibrio* spp. from clinical specimens with a conventional approach has precluded the accumulation of clinical evidence for the treatment of patients suffering from infections with* Desulfovibrio* spp. The currently available automated systems based on biochemical reactions lack a database of properties for identification of this genus, because* Desulfovibrio* spp. are generally nonreactive to biochemical substrates comprising the cards for automated systems [11], meaning that these systems cannot distinguish* Desulfovibrio* spp. from other organisms.

In this report, we describe a case of* Desulfovibrio desulfuricans* bacteremia in an elderly patient suffering from liver abscess. The isolate was confirmed as* D. desulfuricans* using 16S rRNA sequencing. To our knowledge, there has been only one previous report of liver abscess caused by* D. desulfuricans*, an event that was cited in the case report and literature review provided by Hagiwara et al. [5]. However, we believe that the present case report is the first to document the clinical presentation and treatment of such an infection.

## 2. Case Report

**Figure 1:**
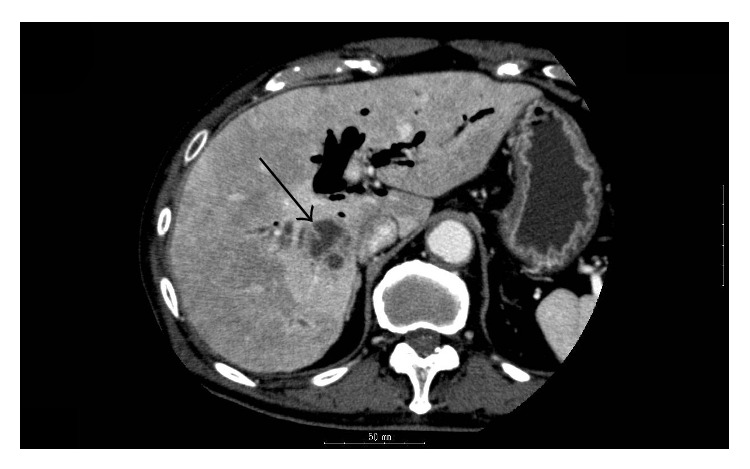
CT scan showing contrast-enhanced lesion in liver (arrow).

An 82-year-old man was admitted to the emergency room because of fever and a sense of abdominal distension. No other gastrointestinal symptoms, such as diarrhea or vomiting, were noted. The patient had a past history of operations by endoscopic sphincterotomy for treatment of cholecystolithiasis. Body temperature was 37.3°C and blood pressure 148/76 mm Hg. Laboratory tests revealed leukocytosis (15500/*μ*L) and elevated level of C-reactive protein (18.97 mg/dL). Gamma-glutamyltransferase (148 U/L) and alkaline phosphatase (507 U/L) were elevated. Alanine aminotransferase (21 IU/L), aspartate aminotransferase (33 IU/L), and total bilirubin (1 mg/dL) were normal, as was renal function. Antibiotic therapy with cefmetazole was started at a dosage of 1 g every 12 hours. On the following day, contrast-enhanced computed tomography (CT) of the abdomen revealed a contrast-enhanced lesion in the liver (Figure 1), indicating liver abscess. Antibiotic therapy was continued without drainage of the abscess because the patient was taking an antiplatelet drug for a previous silent myocardial infarction. On day 2, the patient continued to exhibit an elevated temperature of 39°C. Four sets of blood cultures were taken, and antibiotic therapy was changed to piperacillin-tazobactam at a dosage of 4.5 g every 8 hours. Subsequently, the fever resolved smoothly. On day 11, drip infusion cholecystocholangiography CT showed a reduction in the size of abscess, and the antibiotic was switched to oral amoxicillin-clavulanic acid at a dosage of 1 g/500 mg every day, although microbiological results of blood cultures had not yet been reported. The patient was discharged on day 16 without any symptoms.

**Figure 2:**
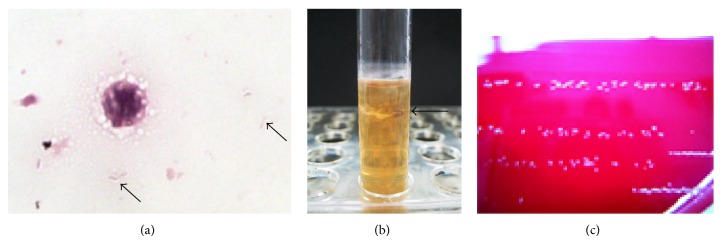
(a) Spiral-shaped bacteria (arrows) in broth from the anaerobic blood culture bottle. Gram stain; ×1000. (b)* D. desulfuricans* (arrow) grown in HK semisolid medium. (c) Colonies of* D. desulfuricans* on Brucella HK agar.

The four sets of blood culture bottles (BacT/ALERT FA and FN) taken on day 2 were cultured with BacT/ALERT 3D (BioMérieux Japan, Tokyo, Japan). Three anaerobic blood culture bottles signaled positive at 4 days after inoculation.* Campylobacter* spp. were initially suspected, because Gram staining revealed gram-negative spiral bacilli (Figure 2(a)). First, broth from the anaerobic bottle was subcultured on sheep blood/chocolate agar (Eiken Chemicals, Tokyo, Japan), incubated at 37°C in 5% CO_2_ or microaerophilically for 4 days, and subcultured on Brucella HK agar (Kyokuto Pharm Ind Co Ltd, Tokyo, Japan) anaerobically at 37°C for 4 days. However, no colony growth was observed with any of these media or conditions. Next, broth from the bottle was subcultured to HK semisolid medium (Kyokuto Pharm Ind Co Ltd, Tokyo, Japan). Growth of an organism was seen in the middle layer of the medium 2 days after inoculation (Figure 2(b)). An aliquot was subcultured on Brucella HK agar and incubated anaerobically again. After 5 days, tiny, translucent, and nonhemolytic colonies were observed on the agar (Figure 2(c)). The growth of this isolate was inhibited by a metronidazole disc (Serotec, Sapporo, Japan). The colonies had a unique fishy smell. Finally, we successfully subcultured the anaerobic bacteria from the blood culture bottle. However, identification of the organism using Rap ID ANA II System (Amco, Tokyo, Japan) did not reveal a specific bacterial species. Therefore, phenotypic testing was performed using standard methods. These tests revealed that the isolate was negative for motility, desulfoviridin, H_2_S production, catalase activity, and indole production, indicating that the isolate was* D. desulfuricans*. Antibiotic susceptibilities were determined by broth microdilution according to the methods of the Clinical Laboratory Standards Institute (CLSI). The minimum inhibitory concentration (MIC) results are shown in Table 1. The isolate was confirmed as* D. desulfuricans* using 16S rRNA sequencing methods.

## 3. Discussion

Although some cases of monobacterial bacteremia with* D. desulfuricans* have been reported [1, 9, 12–14], ours is the first documented case of liver abscess with bacteremia due to* D. desulfuricans* to show how the patient was treated. All previously reported cases of* D. desulfuricans* infection included gastrointestinal symptoms such as diarrhea, nausea, and abdominal distension; this pattern indicates that bacteremia due to* D. desulfuricans* may be induced by bacterial translocation from the intestine. Although* D. fairfieldensis* was suggested to have a higher pathogenic potential than other* Desulfovibrio* spp. [6],* D. desulfuricans* also may exhibit high virulence, as indicated by the ability to cause liver abscess with bacteremia.

The first line antimicrobial agent to treat infectious diseases due to* Desulfovibrio* spp. has not been determined, because few clinical case reports exist to show how to treat such patients with antibiotic therapy. One case report of liver abscess caused by* D. fairfieldensis* reported the antibiotics used. The case was treated with intravenous ampicillin, ceftriaxone, and metronidazole for 1 week, with oral ciprofloxacin and metronidazole administered for a further week [9]. In that case,* D. fairfieldensis* was susceptible to metronidazole and resistant to ampicillin. Studies by Lozniewski et al. and Nakao et al. showed that* Desulfovibrio* spp. exhibit high susceptibilities to carbapenems and metronidazole [15, 16]. Lozniewski et al. suggested that either imipenem or metronidazole should be the agent of choice for treatment of infection with* Desulfovibrio* spp., especially as these species are often isolated from mixed aerobic-anaerobic infections [15]. Although* D. desulfuricans* alone was detected from blood culture in our case, this patient's liver abscess might have been caused by a mix of aerobic and anaerobic bacteria from intestinal flora [17]. Therefore, the patient was treated initially with cefmetazole or subsequently with piperacillin-tazobactam, which are both agents that provide coverage against aerobic and anaerobic bacteria. The antibiotic therapy with piperacillin-tazobactam was successful in treating the patient. The MIC of piperacillin-tazobactam in this strain was relatively low (16 mg/L) compared to those of other* Desulfovibrio* species (e.g., >256 mg/L for* D. fairfieldensis* and* D. piger*) [15, 16]. Thus, the susceptibilities to antimicrobial agents vary depending on the species [15, 16], indicating that the species and the susceptibility to antibiotics should be carefully confirmed when antibiotics are selected to treat infections with* Desulfovibrio* spp.

On the other hand, cefmetazole, which was initially used, was not effective in our case. The MIC of cefmetazole in our isolate was lower than the CLSI susceptibility break point (16 mg/L) used for anaerobes. However, in the case described here, fever and bacteremia persisted until the patient was switched from cefmetazole to piperacillin-tazobactam. Since the susceptibility break point of cefmetazole in anaerobes is higher than that of cefmetazole in the Enterobacteriaceae, higher doses of cefmetazole might have shown efficacy in our patient. Further investigation will be required to determine the appropriate antimicrobial agents for treatment of* Desulfovibrio* spp. infections.

The identification of* Desulfovibrio* spp. by conventional methods is difficult and time-consuming. Ideally, automated systems available in the clinical microbiology laboratory could be used to identify* Desulfovibrio* spp. However, the current available systems, which are based on biochemical tests, are not able to distinguish* Desulfovibrio* spp. from other organisms [11]. New technologies using matrix-assisted laser desorption/ionization time-of-flight (MALDI-TOF) mass spectrometry permit the comparison of the “protein fingerprint” obtained from bacterial cells using a database [18]. This tool has been increasingly applied for the identification of organisms in clinical microbiology laboratories and is expected to shorten the time for the identification of organisms and potentially eliminate the need for biochemical tests. However, the MALDI-TOF databases do not yet include* Desulfovibrio* spp. Inclusion of* Desulfovibrio* spp. in such databases will be required to permit the rapid selection of appropriate antibiotic therapy.

In summary, we report the first case of liver abscess with* D. desulfuricans* bacteremia cured with antibiotic therapy. This case report contributes to the accumulation of clinical data for treatment of patients suffering from infections by* Desulfovibrio* spp.

## Figures and Tables

**Table 1:** The results of antimicrobial susceptibility testing.

Antimicrobial agent	MIC (mg/L)
Ampicillin	<0.5
Piperacillin	16
Amoxicillin-clavulanic acid	<0.75
Piperacillin-tazobactam	16
Cefmetazole	8
Cefoperazone	8
Imipenem	0.5
Panipenem	<0.25
Erythromycin	2
Clindamycin	0.5
Levofloxacin	<0.25
